# Effect of perioperative multistrain probiotics on the CT callus volume fraction at 12 weeks after surgery for closed tibial shaft fracture: a single-center randomized controlled study

**DOI:** 10.3389/fcimb.2026.1778557

**Published:** 2026-08-24

**Authors:** Yongbiao Huang, Xuqiang Zheng, Chengjun Liao, Gang Liu, Mingliang Li

**Affiliations:** 1 Department of Orthopedics, Fujian Provincial Geriatric Hospital, Fuzhou, China; 2 Department of Medical Imaging, Fujian Provincial Geriatric Hospital, Fuzhou, China; 3 Department of Orthopedics, Xinjiang Changji Hui Autonomous Prefecture People’s Hospital, Changji, China

**Keywords:** *Bifidobacterium longum*, bone callus volume fraction, *Lactobacillus rhamnosus* GG, quantitative computed tomography, tibial shaft fracture

## Abstract

**Background:**

Conventional radiographs lack sensitivity for early healing, whereas computed tomography (CT)–derived bone volume fraction (BV/TV) allows quantitative assessment of callus mineralization. Perioperative antibiotics and surgical stress disrupt gut microbiota, potentially impairing fracture healing. Multistrain probiotics may enhance bone repair via immunomodulation and the gut–bone axis.

**Objective:**

To evaluate the effect of perioperative multistrain probiotics (*Lactobacillus rhamnosus* GG plus *Bifidobacterium longum*) on CT-measured BV/TV at 12 weeks.

**Methods:**

This single-center, randomized, double-blind, placebo-controlled trial enrolled 84 patients with closed tibial shaft fractures. The modified intention-to-treat population included participants who received at least one dose and completed follow-up. Probiotics or placebo were administered from the preoperative period through postoperative week 6. The primary outcome was BV/TV at 12 weeks, analyzed using ANCOVA adjusted for age, sex, AO/OTA fracture classification, smoking status, and postoperative interfragmentary gap. Secondary outcomes included time to uncomplicated full weight bearing (defined as ambulation without assistive devices and without pain in the affected limb; analyzed using Kaplan–Meier with the log-rank test), rest-pain visual analog scale (VAS) scores at postoperative weeks 2 and 6, and Radiographic Union Score for Tibial Fractures (RUST) at 12 weeks. CT segmentation reliability was assessed using intraclass correlation coefficients (ICC). Safety outcomes were analyzed using Fisher’s exact test.

**Results:**

The probiotics group demonstrated significantly higher adjusted BV/TV at 12 weeks compared with placebo (Δ = 0.11; 95% CI 0.03–0.19; P = 0.012). In exploratory secondary analyses, for which nominal P values are reported without multiplicity adjustment, time to full weight bearing was shorter (P = 0.041). VAS pain scores were lower at postoperative week 2 (Δ = −0.55; P = 0.047), with no difference at week 6. RUST scores at 12 weeks were higher in the probiotics group (Δ = 0.68; P = 0.019). CT segmentation showed excellent reliability (ICC = 0.96). No significant differences were observed in adverse events and surgical site infection.

**Conclusions:**

Perioperative multistrain probiotics significantly enhanced CT-based bone mineralization at 12 weeks, while exploratory secondary findings suggested earlier functional recovery and improved radiographic healing. These findings warrant further evaluation of probiotics as a potential adjunct to standard fracture care.

## 1 Introduction

Tibial shaft fracture is a common long-bone injury in adults, and the standard treatment is reamed intramedullary nailing (Kang et al., 2021). At 12 weeks postoperatively, patients are typically in a key phase of mineralized callus maturation and transition toward early remodeling; the quality of healing determines weight-bearing recovery and the risk of reoperation (Dailey et al., 2019). Traditionally, healing is monitored by radiographic assessment, although early postoperative radiographs after tibial nailing may show limited interval change and add little information in the absence of clinical concern (Wojahn et al., 2022). CT bone callus volume fraction can objectively quantify the degree of mineralization and is suitable for evaluating short-term structural differences (Wee et al., 2022). Perioperative antibiotics and surgical stress can disrupt the gut microbiota, affecting immune homeostasis and bone remodeling signaling (Nadeem-Tariq et al., 2025). *Lactobacillus rhamnosus* GG and *Bifidobacterium longum* have advantages in maintaining the mucosal barrier, immunomodulation, and short-chain fatty acid metabolism, providing biological feasibility for perioperative application (Mao et al., 2020; Wong et al., 2019). Whether perioperative multistrain probiotics can provide measurable structural benefits in clinical fracture populations still lacks high-quality evidence. Previous studies have mostly focused on osteoporosis or bone mineral density, with few randomized controlled studies targeting postoperative bone repair; imaging endpoints were often based on subjective grading or deferred composite endpoints at later time points, making early mineralization changes difficult to capture (Mehta et al., 2025). Inconsistencies in concomitant medications, rehabilitation cadence, and imaging workflows often cause confounding bias (Bührer et al., 2023). Under a unified perioperative pathway, it is necessary to adopt a double-blind design, quantitative CT endpoints, and quality-controlled measurement procedures to determine whether multistrain probiotics enhance mineralized structure within a clinically relevant healing window and translate into observable functional benefits, while assessing short-term safety. This study aimed to determine whether perioperative multistrain probiotics could improve early fracture healing in patients with closed tibial shaft fractures treated with reamed intramedullary nailing. Specifically, we evaluated whether probiotic supplementation was associated with a higher CT-measured bone callus volume fraction at 12 weeks postoperatively, a time point relevant to mineralized callus maturation and early remodeling. We further examined whether any structural difference was accompanied by concordant changes in clinically meaningful outcomes, including time to uncomplicated full weight bearing, postoperative pain, radiographic healing assessed by RUST, and short-term safety. By focusing on quantitative CT and complementary clinical endpoints, this study sought to clarify the potential value of perioperative microecological intervention in fracture repair practice.

## 2 Materials and methods

### 2.1 Study design

This study was a single-center, randomized, double-blind, placebo-controlled trial. The study sites were the orthopedic ward and imaging center of our hospital. The enrollment period was from 1 January 2024 to 30 April 2025, and the last follow-up was completed on 31 July 31, 2025. All patients underwent standard open reduction and internal fixation for tibial shaft fractures (reamed intramedullary nailing), and, in addition to routine perioperative management, received the multistrain probiotic formulation or a matching placebo; the primary endpoint was assessed once at 12 weeks postoperatively. The study protocol was approved by the institutional medical ethics committee of our hospital (approval number: 20230608). All participants provided written informed consent after receiving a detailed explanation.

### 2.2 Participants

The inclusion criteria were age 18–65 years; unilateral closed tibial shaft fracture (AO/OTA 42A, 42B, or 42C); receipt of reamed intramedullary nail fixation within 72 h after injury; ability to take oral medications postoperatively; and ability to understand and sign informed consent. The exclusion criteria were open fracture or bone defect length greater than 10 mm; pathologic fracture or bone tumor; concomitant multiple injuries with an Injury Severity Score greater than 16; history of inflammatory bowel disease or short bowel syndrome; systemic antibiotic therapy for more than 3 days within the past 14 days; known immunodeficiency or systemic glucocorticoid use during the past 3 months at a prednisone-equivalent dose of 10 mg per day or higher; hemoglobin A1c greater than or equal to 8.5%; estimated glomerular filtration rate less than 45 mL·min^-^¹·1.73 m^-^²; hepatic function Child–Pugh class B or C; planned continuous regular use of nonsteroidal anti-inflammatory drugs for more than 7 days during postoperative weeks 0–6; pregnant or lactation; allergy to any excipient of the preparation; regular supplementation with active probiotics within 14 days before enrollment.

Participants were considered withdrawn or discontinued if they withdrew informed consent; had insufficient compliance (medication intake <80%); developed a severe infection requiring continuous broad-spectrum antibiotic therapy for more than 7 days; experienced a serious adverse event requiring emergency unblinding; exceeded the follow-up time window by more than 14 days and were unable to complete the primary endpoint assessment; or died.

### 2.3 Sample size, randomization, and blinding

The primary endpoint was the difference in BV/TV at 12 weeks postoperatively between the two groups. Published human callus data reported a BV/TV of 0.435 ± 0.077 within 3 months after fracture (Han et al., 2016), providing an external basis for assuming a placebo-group mean of approximately 0.40. To conservatively account for the greater between-patient and CT measurement variability expected in a clinical tibial-fracture cohort, the standard deviation was prespecified as 0.15. A target between-group difference of 0.10 (standardized effect size, 0.67) was prespecified as a clinically relevant structural difference at 12 weeks. Under a two-sample design with two-sided α = 0.05 and 80% power, 36 participants per group were required. Considering a 15% loss to follow-up or nonadherence rate, the target enrollment was set at 42 participants per group, for a total sample size of 84. The sample size was calculated in R 4.3.2 using the prespecified between-group difference, standard deviation, two-sided α = 0.05, and 80% power.

Given the target sample size, a centralized computer-generated randomization sequence allocated participants in a 1:1 ratio to the multistrain probiotic group or the placebo group using variable block randomization with block sizes of 4 and 6, randomly varied to maintain allocation concealment, and stratified by age (<50 years and ≥50 years) and fracture complexity (AO/OTA 42A and 42B/42C). The randomization sequence was generated by independent statisticians and sent to the pharmacy department, and the study team did not have access to the allocation list. The study adopted a double-blind design in which participants, clinical care providers, imaging assessors, and statistical analysts remained blinded until database lock and final analysis. The statistical analysts were not part of the recruitment or clinical care teams. The pharmacy department dispensed individual aluminum-foil sachets that were identical in appearance, weight, and taste according to the randomization number, with labels displaying only the participant ID and administration instructions. Individual unblinding was permitted only when a life-threatening serious adverse event occurred and management decisions depended on treatment allocation. Unblinding was performed by the pharmacy department, with documentation of the time, reason, and responsible person. After trial completion, consolidated unblinding was performed.

### 2.4 Interventions

#### 2.4.1 Multistrain probiotic formulation and dosing regimen

The multistrain probiotic formulation consisted of two standardized strains: *L. rhamnosus* GG (ATCC 53103) (Novonesis, Denmark; LGG-2401-DK-017) and *B. longum* subsp. longum (BB536) (Morinaga Milk Industry Co., Ltd., Japan; BB536-2401-JP-021), formulated as a lyophilized powder. Each sachet contained a total viable count of 1.0 × 10¹^0^ CFU, with the two strains in a 1:1 ratio, equivalent to 5.0 × 10^9^ CFU of each strain per sachet and a total daily dose of 2.0 × 10¹^0^ CFU. The excipient was pharmaceutical-grade maltodextrin. The recommended storage temperature was 2 °C–8 °C. The dosing regimen was one sachet orally twice daily, after breakfast and after dinner, beginning on the third day before surgery and continuing until postoperative day 42 (Nair et al., 2025). If hospital admission occurred less than 3 days before surgery, administration was initiated immediately upon admission and the same daily dose was maintained. In the event of a brief necessary imaging examination or on the day of surgery, a missed dose could be administered once after the participant regained consciousness and oral intake was permitted.

#### 2.4.2 Placebo formulation and dosing regimen

The placebo consisted of maltodextrin powder identical to the probiotic formulation in dose, weight, taste, and packaging but without live bacteria. The frequency and duration of administration were the same as in the experimental group. Routine microbiological culture of the placebo was performed to confirm the absence of cultivable live bacteria.

#### 2.4.3 Standard perioperative management

Perioperative antibiotic prophylaxis consisted of cefazolin 2 g administered by intravenous infusion and completed 30 min before incision, with one additional 2-g dose if the operation lasted more than 3 h; for patients with a severe penicillin allergy, clindamycin 600 mg was administered intravenously (Bunduki et al., 2020). Pain control primarily consisted of stepwise analgesia with acetaminophen and opioids, avoiding routine long-term use of nonsteroidal anti-inflammatory drugs; celecoxib 200 mg up to twice daily for no more than three consecutive days was permitted as rescue medication, and the cumulative dose was recorded (Murphy et al., 2023). Thromboprophylaxis consisted of a standard low-molecular-weight heparin regimen. The surgical procedure was standardized as a reamed, locked intramedullary nail fixation and was performed by a group of qualified lead surgeons. The weight-bearing protocol of non-weight bearing during the first two postoperative weeks, partial weight bearing during postoperative weeks 3–6, and a gradual transition to full weight bearing during postoperative weeks 7–12 (Warmerdam et al., 2023); the progression was uniformly determined and recorded by the same rehabilitation team based on clinical and imaging assessments.

### 2.5 Observation time points and follow-up procedures

Visit time points were scheduled for the preoperative assessment (preoperative days −3 to −1), postoperative day 1, postoperative day 14 (± 2 days), postoperative day 42 (± 5 days), and postoperative week 12 (± 7 days). At each visit, vital signs, incision status, medications, and adverse events were recorded. If a serious or life-threatening adverse event occurred between scheduled visits, participants were instructed to seek immediate emergency care and notify the study team without delay. Upon becoming aware of the event, the investigators initiated an urgent safety assessment and reporting, and emergency unblinding was performed when clinical management depended on treatment allocation. Compliance was verified by counting returned empty sachets and cross-checking them against the daily medication cards; missed doses for more than three consecutive days were specifically flagged. During follow-up, weekly telephone follow-up was conducted to record exposure to antibiotics and nonsteroidal anti-inflammatory drugs. At postoperative week 12, a limited-range CT scan was performed, and the primary endpoint was assessed.

### 2.6 Outcomes

The primary outcome was CT bone callus volume fraction (BV/TV) at 12 weeks postoperatively (Morgan et al., 2009), defined as the ratio of the volume of mineralized bone with a density above a prespecified threshold within a prespecified three-dimensional region of interest to the total volume of that region. Results were expressed as a dimensionless decimal and were also converted to percentages for reporting. Specific measurement methods are detailed in *Section 2.7*.

Secondary outcomes included time to uncomplicated full weight bearing (defined as the first achievement of ambulation without assistive devices and without pain in the affected limb, with the event date prospectively recorded by the rehabilitation team during scheduled visits and interval follow-up contacts); the incidence of surgical site infection within 6 weeks postoperatively (according to the Centers for Disease Control and Prevention criteria for surgical site infection) (Mangram et al., 1999), with infections occurring after 6 weeks and up to postoperative week 12 recorded during safety follow-up and reflected in other safety outcomes as appropriate but not counted in this prespecified endpoint; rest-pain visual analog scale at postoperative weeks 2 and 6 (scale range 0–10) (Maarj et al., 2022); Radiographic Union Score for Tibial Fractures (RUST, using the four-cortex method) at postoperative week 12 (Kooistra et al., 2010); the incidence of unplanned reoperation within 12 weeks postoperatively; and the incidence and severity grading of intervention-related adverse events.

### 2.7 CT examination and measurement of callus volume fraction

#### 2.7.1 CT scanning parameters and reconstruction protocol

A 64-detector-row Revolution EVO CT scanner (GE HealthCare, USA) was used. Participants were scanned in the supine position, with both lower limbs maintained in the neutral position. The scanning range extended 40 mm proximally and 40 mm distally from the fracture line, for a total length of 80 mm. The tube voltage was 120 kVp, the reference current was 100 mAs, the pitch was 0.8, and the detector collimation was 64 mm × 0.625 mm. The reconstruction slice thickness was 0.625 mm with a reconstruction interval of 0.5 mm, using the Bone Plus reconstruction kernel and a 512 × 512 reconstruction matrix, with Smart MAR metal artifact reduction enabled. The target dose-length product was controlled at 200 mGy·cm or lower. All examinations were performed by the same imaging team under a unified protocol.

#### 2.7.2 Callus segmentation method and BV/TV calculation workflow

Semi-automatic segmentation was performed using 3D Slicer (version 5.2). First, within the 80-mm scanning volume, the outer callus margin was manually delineated on every axial slice and reviewed in the coronal and sagittal planes. Native cortical bone and the medullary cavity were segmented separately and subtracted; interfragmentary callus contiguous with the periosteal callus was included, whereas surrounding soft tissue, the intramedullary nail, and locking screws were excluded. The resulting mask constituted the callus region of interest. A fixed-threshold method was then used to identify mineralized bone tissue, with the threshold set at 180 HU (Sewify et al., 2025). The threshold was calibrated before study initiation by scanning a phantom and 10 pilot cases and remained unchanged throughout the study. For metallic implant regions, all voxels above 3,000 HU were first masked using intensity thresholding. The metal mask was then isotropically expanded by 3 mm and excluded from both BV and TV to prevent the metal and adjacent streak artifacts from being classified as mineralized tissue (Karageorgos et al., 2024). BV was defined as the sum of the volumes of all voxels above 180 HU within the valid callus region of interest after metal-mask exclusion; TV was defined as the corresponding total valid callus region-of-interest volume (Nadkarni et al., 2025). BV/TV was calculated as BV divided by TV, with results presented to two decimal places and also provided as percentages. All computations were completed on the same workstation, and the export scripts and parameter files were archived for review.

### 2.8 Assessor agreement and quality control

Two assessors with more than 5 years of experience in musculoskeletal imaging participated in the image assessments under complete blinding. One designated assessor completed segmentation and quantitative measurements for all cases, while the second assessor independently repeated the procedure for the first 20 enrolled participants and reviewed the segmentation masks for the remaining cases. Any discrepancies in callus boundary delineation or metal-mask exclusion were resolved by consensus. Unresolved cases were adjudicated by a third senior assessor blinded to group allocation, and the consensus or adjudicated segmentation was used in the final analysis. In this 20-participant subset, after both assessors had completed their initial independent measurements, inter-rater agreement was assessed by comparing their measurements. The intraclass correlation coefficient (ICC[2,1]) was calculated using a two-way random-effects model with absolute agreement for single measurements. If the ICC was below 0.90, the prespecified contingency procedure required a third senior assessor, blinded to group allocation, to convene a consensus meeting and establish standardized operating procedures, after which the inter-rater assessment would be repeated. A monthly cross-check of the scanning protocol and software version was performed, and a fixed phantom was used to monitor HU stability. Enrollment was paused, and calibration performed if HU drift exceeded ±10 HU.

### 2.9 Statistical analysis methods

The primary analysis used the modified intention-to-treat (mITT) set, which included all randomized participants who received at least one dose of the allocated intervention and had at least one medication record. This prespecified analysis set was used because three randomized participants did not receive the allocated intervention. The per-protocol analysis served as a supportive sensitivity analysis and included only participants with treatment compliance of 80% or higher and no major protocol deviations. Continuous variables were summarized as the mean and standard deviation or the median and interquartile range, according to their distribution, and categorical variables as counts and percentages. Statistical analyses were performed using R 4.3.2.

Between-group comparison of the primary endpoint used an analysis of covariance (ANCOVA) model with treatment group as a fixed factor and the prespecified covariates of age, sex, AO/OTA classification (42A vs 42B/42C), smoking status (current vs non-current), and the immediate postoperative initial interfragmentary gap (mm), defined as the mean of the measurements obtained on AP and lateral radiographs and analyzed as a single summary value. Least-squares mean differences and 95% confidence intervals were reported. Model residuals were tested for normality and homoscedasticity, and robust standard errors were applied when necessary. A two-sample t test was used as a sensitivity analysis, and a rank-transformed ANCOVA was used for verification when distributions were skewed. When the primary endpoint was missing and the proportion of missing data did not exceed 20%, multiple imputation (chained equations, 50 imputations, including treatment group, stratification factors, and key covariates) was employed, and estimates were combined using Rubin’s rules. If the proportion exceeded 20%, the distribution of reasons for missingness was presented in the report, and extreme-scenario analyses were performed. For secondary outcomes, time to full weight bearing was displayed using Kaplan–Meier curves and compared with the log-rank test. The incidences of surgical site infection and unplanned reoperation were compared using Fisher’s exact test. Rest-pain VAS scores at postoperative weeks 2 and 6 were analyzed separately using ANCOVA, and RUST scores at postoperative week 12 were analyzed using ANCOVA, with the same covariates used in the primary endpoint analysis. All tests were two-sided with a significance level of 0.05. No multiplicity adjustment was performed, and results for secondary outcomes were interpreted as exploratory.

## 3 Results

### 3.1 Participant enrollment and baseline characteristics

**Figure 1:**
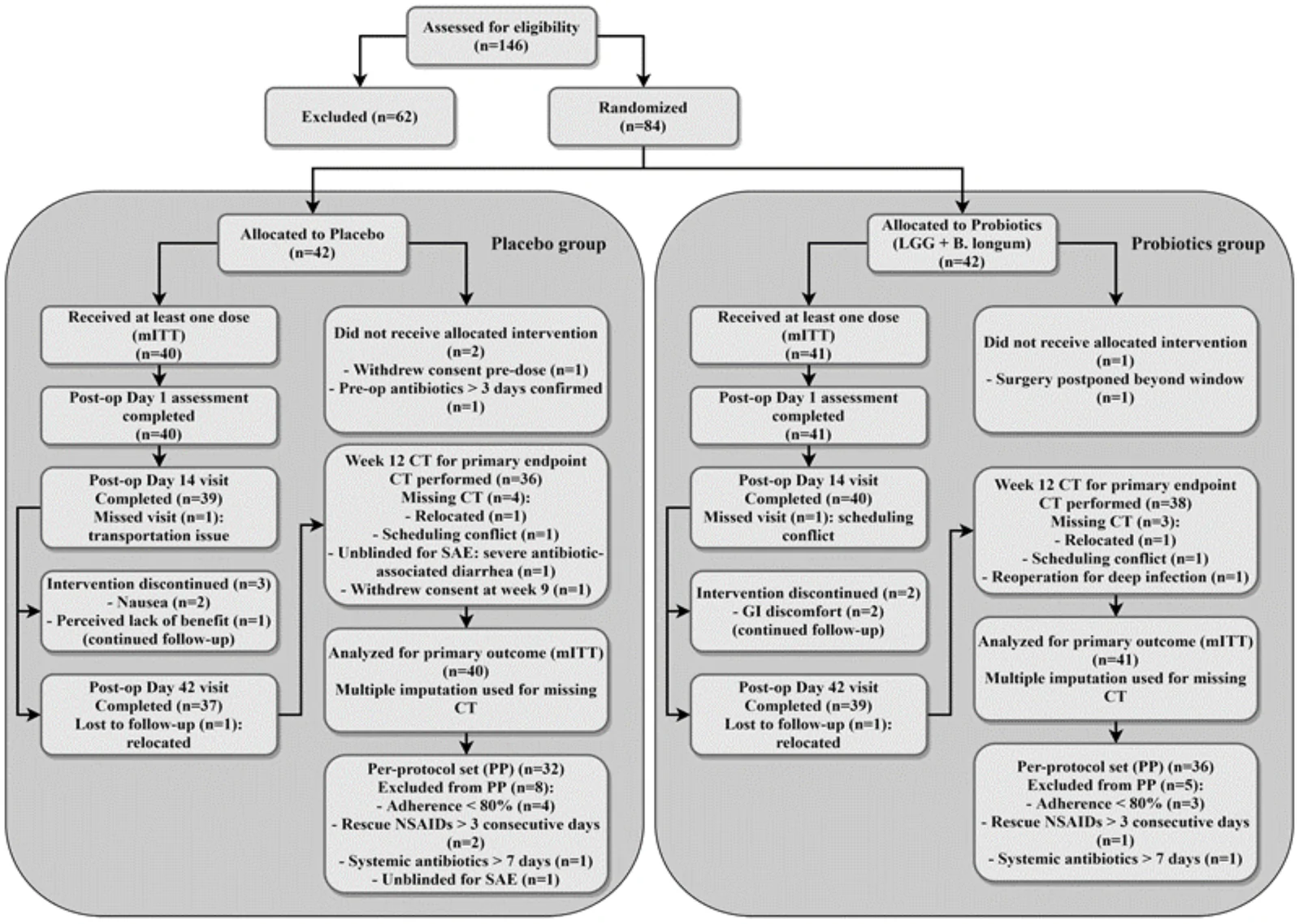
Participant flow diagram (CONSORT).

A total of 146 patients with tibial shaft fractures were assessed for eligibility, and 84 were randomized (42 to the multistrain probiotic group and 42 to the placebo group). In the multistrain probiotic group, 41 participants received the allocated intervention and were included in the mITT set; in the placebo group, 40 participants received the allocated intervention and were included in the mITT set. The remaining three randomized participants did not receive the allocated intervention and were therefore not included in the prespecified mITT set. At 12 weeks postoperatively, CT evaluation was completed in 38 and 36 participants in the multistrain probiotic and placebo groups, respectively. All 81 participants in the mITT set were retained in the primary analysis, with multiple imputation applied for missing CT values. Because of insufficient treatment compliance or protocol deviations related to concomitant medications, the PP set included 36 and 32 participants, respectively (**Figure 1**). After randomization, the demographic and clinical baseline characteristics were similar between the two groups. The mean age was approximately 41 years, and the sex distribution and proportions of AO/OTA classifications were comparable. The stratification factors (<50/≥50 years, 42A and 42B/42C) were similarly distributed between the groups. Current smokers accounted for 32.14%, and the mean immediate postoperative interfragmentary gap measured on radiographs was approximately 1.95 mm (**Table 1**). **Table 1** is presented for descriptive purposes only, and no between-group significance testing was performed.

**Table 1:** Balance of baseline characteristics and stratification factors between the two groups $\left(\bar{x}\pm s)/(n,\%\right)$.

Variables	Multistrain probiotic group (n = 42)	Placebo group (n = 42)	Overall (n = 84)
Age (years)	41.28 ± 11.37	40.93 ± 11.62	41.11 ± 11.43
Male	27 (64.29%)	26 (61.90%)	53 (63.10%)
Female	15 (35.71%)	16 (38.10%)	31 (36.90%)
AO/OTA classification			
42A	18 (42.86%)	17 (40.48%)	35 (41.67%)
42B	15 (35.71%)	15 (35.71%)	30 (35.71%)
42C	9 (21.43%)	10 (23.81%)	19 (22.62%)
Stratified age			
<50 years	29 (69.05%)	28 (66.67%)	57 (67.86%)
≥50 years	13 (30.95%)	14 (33.33%)	27 (32.14%)
Stratified complexity			
42A	18 (42.86%)	17 (40.48%)	35 (41.67%)
42B/42C	24 (57.14%)	25 (59.52%)	49 (58.33%)
Current smoking	14 (33.33%)	13 (30.95%)	27 (32.14%)
Immediate postoperative X-ray initial interfragmentary gap (mm)	1.93 ± 0.67	1.96 ± 0.70	1.95 ± 0.68

### 3.2 Intervention implementation and quality control

Overall compliance with the intervention was high in both groups, with median medication completion rates above 90% and approximately 90% of participants achieving ≥80% completion. During postoperative weeks 0–12, the cumulative duration of rescue NSAID use and additional systemic antibiotic therapy was low and similar between the groups, suggesting that perioperative concomitant medication exposure was well balanced (**Table 2**). In the first 20 enrolled participants, two assessors with musculoskeletal imaging experience independently performed CT callus segmentation and calculated BV/TV under complete blinding. Inter-rater agreement was assessed using the intraclass correlation coefficient [ICC(2,1)] based on a two-way random-effects model with absolute agreement. The ICC for BV/TV was 0.96 (95% CI, 0.92–0.99), exceeding the prespecified acceptable threshold (ICC≥0.90).

**Table 2:** Intervention compliance and perioperative concomitant medication exposure (n,%)/M[IQR].

Indicator	Multistrain probiotic group (mITT, n = 41)	Placebo group (mITT, n = 40)	Overall (mITT, n = 81)
Medication completion rate (%)	92.34[87.12–97.58]	91.47[86.03–96.21]	91.95[86.78–97.24]
Number achieving ≥80% completion	38(92.68%)	36(90.00%)	74(91.36%)
Cumulative days of rescue NSAIDs (postoperative 0–12 weeks)	1[0–2]	2[0–3]	1[0–3]
Cumulative days of additional systemic antibiotics (postoperative 0–12 weeks)	0[0–1]	0[0–2]	0[0–2]

mITT: participants who, after randomization, took at least one dose and had medication records. Rescue NSAIDs and additional systemic antibiotics are exposure quantification metrics recorded during follow-up, used for quality control and contextual interpretation, and are not included in primary effect inference.

### 3.3 Primary outcome: CT callus volume fraction

**Figure 2:**
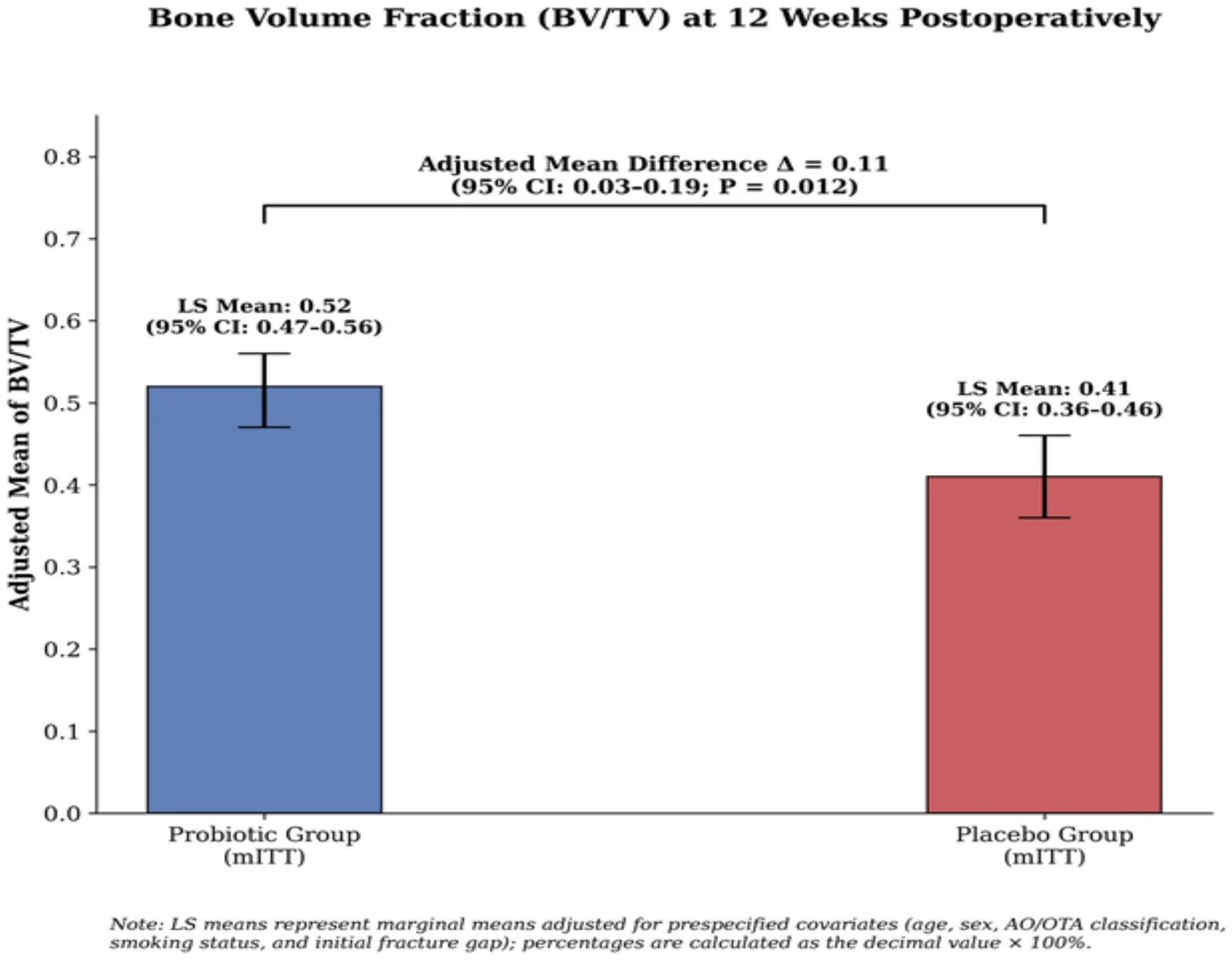
Bone volume fraction (BV/TV) at 12 weeks postoperatively.

Using analysis of covariance (ANCOVA) adjusted for age, sex, AO/OTA classification, smoking status, and the immediate postoperative initial interfragmentary gap, the adjusted LS mean BV/TV at 12 weeks postoperatively was significantly higher in the multistrain probiotic group (0.52) than in the placebo group (0.41) (Δ = 0.11, 95% CI, 0.03–0.19, P = 0.012) (adjusted Cohen’s d = 0.58) (**Figure 2**).

### 3.4 Secondary outcomes

**Figure 3:**
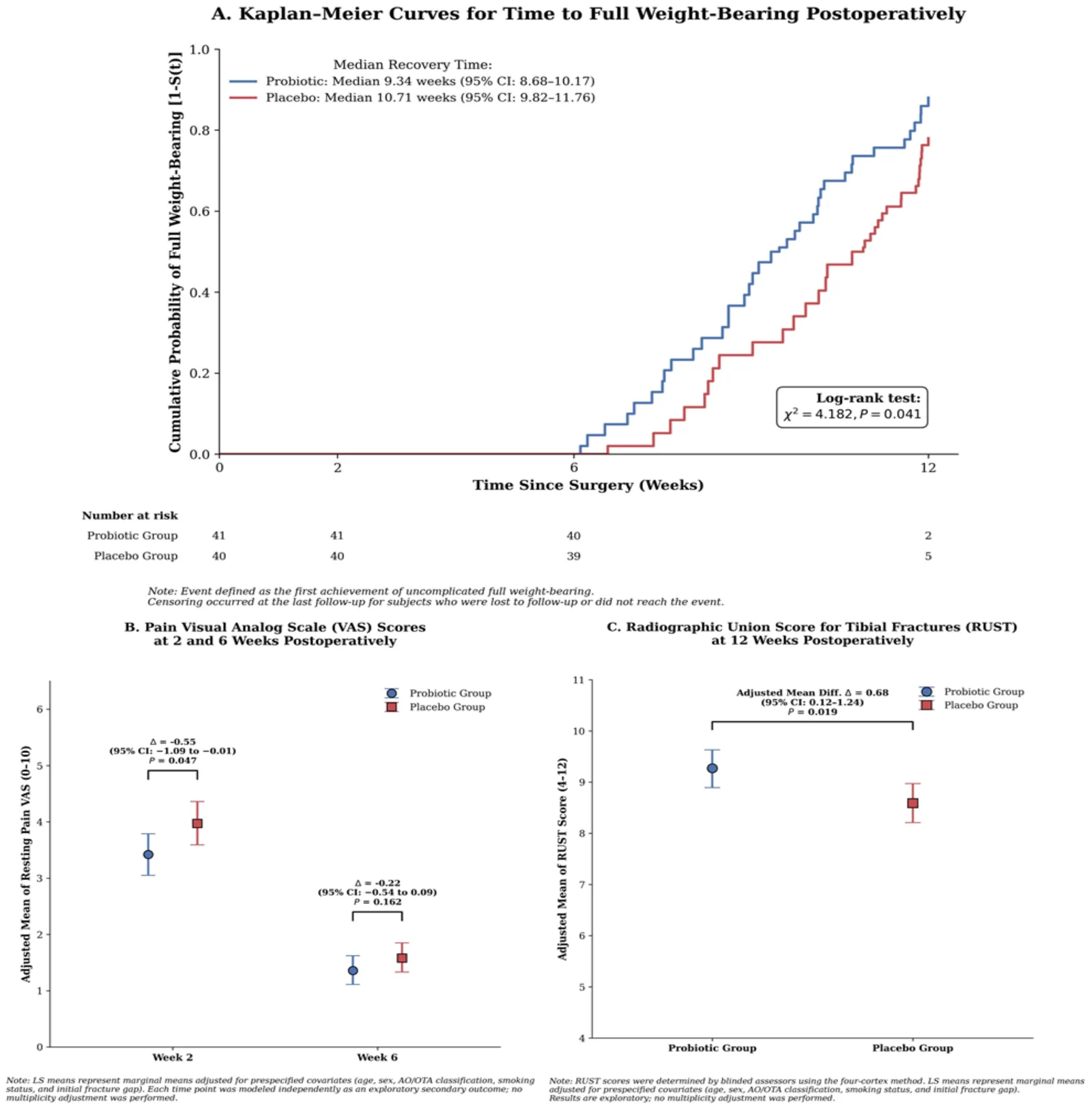
Postoperative fracture healing and functional outcomes. **(A)** Bone volume fraction (BV/TV) at 12 weeks postoperatively. **(B)** Pain visual analog scale (VAS) scores at 2 and 6 weeks postoperatively. **(C)** Radiographic union score for tibial fractures (RUST) at 12 weeks postoperatively.

All secondary-outcome analyses were exploratory, and the reported P values and 95% CIs were nominal and unadjusted for multiplicity. Time to uncomplicated full weight bearing was estimated using the Kaplan–Meier method, and the time-to-event distributions were compared using the log-rank test. Participants in the multistrain probiotic group achieved full weight bearing earlier than those in the placebo group (median, 9.34 weeks [95% CI, 8.68–10.17] vs 10.71 weeks [95% CI 9.82–11.76]; events: 36/41 vs 31/40; P = 0.041) (**Figure 3A**). Using ANCOVA with the prespecified covariates, the adjusted LS mean rest-pain VAS score at postoperative week 2 was lower in the multistrain probiotic group than in the placebo group (Δ = −0.55, 95% CI −1.09 to −0.01, P = 0.047) (adjusted Cohen’s d = −0.45). The between-group difference decreased at postoperative week 6 and did not reach statistical significance (Δ = −0.22, 95% CI, −0.54 to 0.09, P = 0.162) (adjusted Cohen’s d = −0.31) (**Figure 3B**). Using ANCOVA with the prespecified covariates, the adjusted LS mean RUST at postoperative week 12 was higher in the multistrain probiotic group than in the placebo group (Δ = 0.68, 95% CI, 0.12–1.24, P = 0.019) (adjusted Cohen’s d = 0.54) (**Figure 3C**).

### 3.5 Safety

Safety outcomes were compared between groups using Fisher’s exact test. Within 6 weeks postoperatively, there was no statistically significant difference between the groups in the incidence of surgical site infection. During the study period, there was no statistically significant difference in intervention-related adverse events, and all such events were mild, with no serious intervention-related adverse event observed. Within 12 weeks postoperatively, there was no statistically significant difference between the groups in the incidence of unplanned reoperation (all P >0.05) (**Table 3**).

**Table 3:** Safety outcomes (n, %).

Event type	Multistrain probiotic group (n = 41)	Placebo group (n = 40)	Risk difference, percentage points (95% CI)	P (Fisher)
Surgical site infection (within 6 weeks postoperatively)	0(0.00%)	1(2.50%)	−2.50 (−12.88 to 6.31)	0.494
Adverse events related to the intervention (during the study period)	2(4.88%)	2(5.00%)	−0.12 (−12.16 to 11.71)	1.000
Unplanned reoperation (within 12 weeks postoperatively)	1(2.44%)	0(0.00%)	2.44 (−6.55 to 12.60)	1.000

Surgical site infection was defined and determined according to CDC criteria. Intervention-related adverse events were judged by blinded investigators as “possibly related” or “likely related.”.

## 4 Discussion

After covariate adjustment, CT callus BV/TV at 12 weeks remained higher in the multistrain probiotic group than in the placebo group, and the difference persisted after controlling for smoking status, fracture classification, and the immediate postoperative interfragmentary gap, suggesting that perioperative supplementation with *L. rhamnosus* GG combined with *B. longum* increased the proportion of mineralized callus volume. At this time point, mineralization and remodeling are accelerating, and the structural gain may provide a more stable callus foundation for progressive weight bearing during rehabilitation. High treatment compliance and similar exposure to rescue NSAIDs and additional systemic antibiotics reduced the likelihood that differences in concomitant medication use explained the observed outcomes. The use of a fixed HU threshold, standardized artifact handling, and blinded dual-assessor segmentation with high inter-rater agreement supports the conclusion that the observed differences likely reflect true healing rather than measurement error. After intramedullary nailing for tibial shaft fractures, antibiotic prophylaxis, surgical stress, and restricted activity may induce short-term gut microbiota imbalance and systemic inflammatory fluctuations. Upregulation of inflammatory mediators can suppress osteogenesis and promote osteoclast activity, thereby affecting the transition from soft callus to hard callus (Zhao et al., 2021; Maruyama et al., 2020). The complementary effects of the two strains may improve intestinal barrier function, reduce endotoxin translocation, increase short-chain fatty acid production, and optimize mineral absorption. Short-chain fatty acids may inhibit osteoclast activation through immunomodulation, thereby promoting mineral deposition (Zaiss et al., 2019). Previous clinical studies have mostly relied on radiographic grading or time to healing. Although validated radiographic tools such as RUST have good reliability, they are less sensitive than CT-based quantitative measures for detecting early mineralization differences. Three-dimensional CT-derived BV/TV provides complementary structural information and should not be interpreted as equivalent to radiographic healing (Nicholson et al., 2021). Probiotic research in bone metabolism suggests that the gut microbiota can modulate bone remodeling signaling (Lyu et al., 2023), but randomized evidence using structural endpoints in postoperative trauma populations remains limited. Moreover, the lack of concurrent microbiota and inflammatory marker measurements means that the proposed mechanisms require further verification in future studies.

The present findings should be interpreted within the broader context of biological approaches to fracture healing. In complex tibial injuries and cases of nonunion, several adjunctive strategies have been investigated, including teriparatide, platelet-rich plasma, hyperbaric oxygen therapy, external fixation-based reconstruction, and bone reconstruction techniques. Although these approaches are generally reserved for delayed healing, nonunion, bone loss, or severe trauma, they support the principle that modulation of the biological healing environment may complement mechanical stability. Perioperative probiotics may represent a noninvasive, low-risk adjunct that acts through distinct gut–bone and immune-modulatory pathways. Future studies should compare or combine microbiota-targeted approaches with established biological therapies and include biomarkers of inflammation, bone turnover, and gut microbiota composition.

Recent advances in orthopedic research have increasingly focused on biological enhancement strategies to accelerate fracture healing, particularly in the management of tibial fractures, delayed union, nonunion, and complex bone reconstruction. A recent systematic review by Meccariello et al. (2026) highlighted emerging reconstructive approaches for severe long-bone trauma and emphasized the importance of biological augmentation to improve bone regeneration and functional recovery. Likewise, Rollo et al. (2021) demonstrated that combining teriparatide with the Ilizarov technique significantly enhanced bone healing and union rates in septic tibial nonunion, whereas Meccariello et al. (2025) reported improved fracture healing and Radiographic Union Score for Tibial Fractures (RUST) following adjuvant teriparatide therapy in tibial plafond injuries. Furthermore, Rollo et al. (2020) showed that biological adjuncts such as platelet-rich plasma and hyperbaric oxygen therapy can accelerate callus formation and promote healing in aseptic tibial nonunion. Compared with these regenerative interventions, perioperative probiotic supplementation represents a fundamentally different yet complementary biological strategy. Rather than directly stimulating osteogenesis through pharmacological or surgical means, probiotics may enhance fracture repair by modulating the gut–bone axis, reducing systemic inflammation, improving mineral absorption, and supporting favorable bone remodeling. Although the present study evaluated uncomplicated closed tibial shaft fractures rather than delayed union or nonunion, our findings suggest that microbiota-targeted therapy may represent a safe, inexpensive, and noninvasive adjunct to conventional orthopedic management. Future multicenter studies should investigate whether combining probiotics with established biological enhancement strategies, including anabolic agents and regenerative therapies, could further improve healing outcomes in patients with delayed union, nonunion, and complex reconstructive procedures.

The time-to-event distribution for uncomplicated full weight bearing was shifted earlier in the multistrain probiotic group, suggesting that the structural healing advantage could translate into meaningful functional benefits during rehabilitation. Full weight bearing is a key milestone for patients with tibial shaft fracture because it facilitates restoration of gait and return to work. Achieving it earlier may reduce muscle weakness and joint stiffness associated with prolonged protected weight bearing. A nominal between-group difference in rest pain was observed at postoperative week 2 but not at week 6. Because no multiplicity adjustment was applied, this finding should be interpreted as an exploratory early signal rather than confirmatory evidence of an analgesic effect. The nominally higher RUST score at 12 weeks was directionally concordant with the increase in CT-measured callus bone volume fraction (BV/TV), providing supportive rather than confirmatory evidence of accompanying cortical healing. Micromotion at the fracture site during the transition to weight bearing can prolong the inflammatory phase and amplify pain. Probiotics may improve intestinal barrier function, reduce endotoxin-related immune activation, and modulate the balance between osteoclast and osteoblast activity, thereby promoting more complete hard callus formation, increasing early load bearing capacity, and reducing pain (Nishimura et al., 2025). Previous studies generally suggest that pain reduction and progression to full weight bearing after intramedullary nailing occur primarily within the first several weeks to three months postoperatively. The observed differences were concentrated within this early period, consistent with the intervention schedule that began perioperatively and continued through postoperative week 6 (Tamburini et al., 2023). The literature also indicates that RUST correlates with clinical weight-bearing capacity but has limited sensitivity for detecting early differences. Therefore, the directional concordance of CT, RUST, and functional outcomes strengthens the internal consistency of the findings but does not establish confirmatory evidence for the secondary endpoints (Atwan and Schemitsch, 2020). Because secondary outcomes were not adjusted for multiplicity, they should be interpreted as exploratory, supportive signals rather than independent evidence of efficacy.

The high quality of intervention implementation and outcome measurement provided a reliable basis for the main conclusions. Treatment compliance remained high in both groups, indicating adequate perioperative probiotic exposure and making it unlikely that the treatment effect on the primary endpoint was attenuated by insufficient dosing. During follow-up, exposure to rescue NSAIDs use and additional systemic antibiotic therapy was low and similar between the groups. Together with standardized perioperative antibiotic prophylaxis, a unified rehabilitation weight-bearing protocol, and the double-blind design, this made the observed imaging and functional differences less likely to be explained by concomitant medication use or treatment bias. CT callus segmentation achieved high inter-rater agreement under blinded conditions, reducing random measurement error associated with metal artifacts and threshold-based segmentation and improving the reproducibility of the quantitative BV/TV endpoint and the stability of the effect estimates (Singh et al., 2022). In terms of safety, there were no between-group differences in short-term surgical site infection, i9ntervention-related adverse events, or unplanned reoperation, supporting the feasibility of perioperative use of the multistrain probiotic formulation in immunocompetent patients with closed tibial shaft fractures undergoing standard intramedullary nailing. Previous perioperative probiotic studies have mainly focused on rare such as bacteremia in immunosuppressed or severely comorbid populations (Mikucka et al., 2022). The strict exclusion criteria in this study and the observed favorable safety profile are consistent with such these findings and suggest that broader application remain limited to appropriately selected populations with careful perioperative monitoring until additional evidence becomes available. The study population was well-defined, the dosing regimen was standardized without increasing nursing workload, and the intervention was practical to implement. Single-center procedural consistency helped reduce the impact of variations in surgical technique and imaging parameter drift on callus quantification, thereby providing a reproducible framework for future multicenter validation.

### 4.1 Limitations

This was a single-center study with a moderate sample size. The surgical team, rehabilitation pathway, and imaging workflow were relatively uniform, which helps control variability; however, differences in antibiotic prophylaxis, analgesic strategies, and imaging equipment across hospitals may affect generalizability of the findings. Therefore, multicenter replication and external validation are warranted. The inclusion and exclusion criteria excluded patients with open fractures, severe comorbidities, or immunocompromising conditions; therefore, the findings are most applicable to relatively low-risk patients with closed tibial shaft fractures undergoing standard intramedullary nailing. Because enrollment was limited to adults aged 18–65 years, the findings should not be directly extrapolated to patients older than 65 years or to populations with osteoporotic or fragility fractures. Follow-up was limited to 12 weeks. Therefore, later-stage bone remodeling, delayed union, nonunion, malunion, and longer-term functional and quality-of-life outcomes were not evaluated. It remains unknown whether the early structural benefit persists at 6 or 12 months or translates into clinically meaningful differences. Fecal microbiota, short-chain fatty acids, and inflammatory markers were not assessed, nor were dietary composition, vitamin D intake, or calcium supplementation systematically recorded, making it impossible to verify the associations between intestinal changes and quantitative callus indices at the individual level or to account for potential confounding factors. Although a standardized criterion was used to determine full weight bearing, rehabilitation implementation relied primarily on follow-up records without objective monitoring. In addition, the VAS is a subjective measure and may be influenced by psychological expectations and social factors. BV/TV was calculated using a fixed HU threshold and a metal artifact processing script, with good within-institution consistency; however, recalibration would be required under different scanning parameters or reconstruction algorithms. In addition, probiotic viable counts were not retested for each batch during the study period, and a small proportion of missing primary endpoint data was handled using multiple imputation, which may still have introduced uncertainty.

## 5 Conclusions

Perioperative multistrain probiotic supplementation (LGG + *B. longum*) in patients with closed tibial shaft fractures treated with reamed intramedullary nailing increased CT-measured bone callus volume fraction at 12 weeks compared with placebo. The exploratory secondary findings, reported without multiplicity adjustment, were directionally consistent with earlier achievement of uncomplicated full weight bearing, improved radiographic healing as assessed by RUST, and reduced early postoperative pain. High treatment compliance, a safety profile comparable to that of the control group, and high consistency of imaging measurements suggest that this strategy is feasible within standard perioperative care and warrants further clinical evaluation. The primary structural finding, together with the directionally concordant exploratory outcomes, supports further multicenter evaluation of whether perioperative microecological optimization may facilitate early mineralization and functional recovery. However, routine incorporation into standard treatment workflows requires confirmatory evidence.

## Data Availability

The original contributions presented in the study are included in the article/supplementary material. Further inquiries can be directed to the corresponding author.
